# Development and validation of a prognostic nomogram for predicting overall survival in patients with primary bladder sarcoma: a SEER-based retrospective study

**DOI:** 10.1186/s12894-021-00929-x

**Published:** 2021-11-25

**Authors:** Shijie Li, Xuefeng Liu, Xiaonan Chen

**Affiliations:** Department of Urology, Shengjing Hospital of China Medical University, 36 Sanhao Street, Shenyang, 110004 Liaoning People’s Republic of China

**Keywords:** Primary bladder sarcoma, Nomogram, Prognosis, Survival analysis

## Abstract

**Background:**

Primary bladder sarcoma (PBS) is a rare malignant tumor of the bladder with a poor prognosis, and its disease course is inadequately understood. Therefore, our study aimed to establish a prognostic model to determine individualized prognosis of patients with PBS.

**Patients and Methods:**

Data of 866 patients with PBS, registered from 1973 to 2015, were extracted from the surveillance, epidemiology, and end result (SEER) database. The patients included were randomly split into a training (n = 608) and a validation set (n = 258). Univariate and multivariate Cox regression analyses were employed to identify the important independent prognostic factors. A nomogram was then established to predict overall survival (OS). Using calibration curves, receiver operating characteristic curves, concordance index (C-index), decision curve analysis (DCA), net reclassification improvement (NRI) and integrated discrimination improvement (IDI), the performance of the nomogram was internally validated. We compared the nomogram with the TNM staging system. The application of the risk stratification system was tested using Kaplan–Meier survival analysis.

**Results:**

Age at diagnosis, T-stage, N-stage, M-stage, and tumor size were identified as independent predictors of OS. C-index of the training cohort were 0.675, 0.670, 0.671 for 1-, 3- and 5-year OS, respectively. And that in the validation cohort were 0.701, 0.684, 0.679, respectively. Calibration curves also showed great prediction accuracy. In comparison with TNM staging system, improved net benefits in DCA, evaluated NRI and IDI were obtained. The risk stratification system can significantly distinguish the patients with different survival risk.

**Conclusion:**

A prognostic nomogram was developed and validated in the present study to predict the prognosis of the PBS patients. It may assist clinicians in evaluating the risk factors of patients and formulating an optimal individualized treatment strategy.

**Supplementary Information:**

The online version contains supplementary material available at 10.1186/s12894-021-00929-x.

## Introduction

Primary bladder sarcoma (PBS) is a very rare malignant tumor, accounting for less than 0.5% of all bladder tumors. The 5-year survival rate is 10–35% [1]. Some subtypes of PBS show a high tendency toward distant metastasis and are associated with a shorter survival [2]. Published studies on PBS are scarce all over the world, and most of the cases are reported in the form of case reports for a certain subtype of PBS [3–5].

In view of the scarcity of PBS, the natural history of this disease is not well known. Nevertheless, its relationship with schistosomiasis [6], cyclophosphamide therapy [7, 8], and radiotherapy [9] has been documented. Leiomyosarcoma is the most common type of bladder sarcoma in adults and it is reported that the incidence rate of bladder leiomyosarcoma may increase due to an increase in the number of patients undergoing chemo or radiotherapy [10].

Insufficient understanding makes the diagnosis and treatment of PBS challenging in daily practice. Due to the low incidence rate, the treatment of PBS is largely empirical. Radical cystectomy remains the mainstay of treatment for non-metastatic PBS, and bilateral pelvic lymph node dissection is recommended because of the high risk of metastasis to the pelvic lymph nodes [11]. Transurethral resection alone is generally not recommended, except for very small lesions [3]. Chemo and radiotherapy are viable treatment options as well, and are usually used in the comprehensive treatment of rhabdomyosarcoma or high-grade metastatic leiomyosarcoma [12, 13]. Radiotherapy is also chosen in case of positive surgical margins or suspicion of residual tumor [14].

For rare tumors such as PBS, single center studies often have poor predictive power due to the small number of patients. Therefore, using a population-based cancer database to assess the clinical characteristics and prognosis is a reasonable way to acquire better understanding of this rare disease. In this study, we used the Surveillance, Epidemiology, and End Results (SEER) database (https://seer.cancer.gov/) to identify the prognostic factors and construct a nomogram for PBS patients. To the best of our knowledge, this is the first study using the SEER database to examine the clinical features of PBS. This study aimed to establish a predictive model to better understand the survival outcomes of PBS at a population level.

## Material and methods

### Patients

**Fig. 1:**
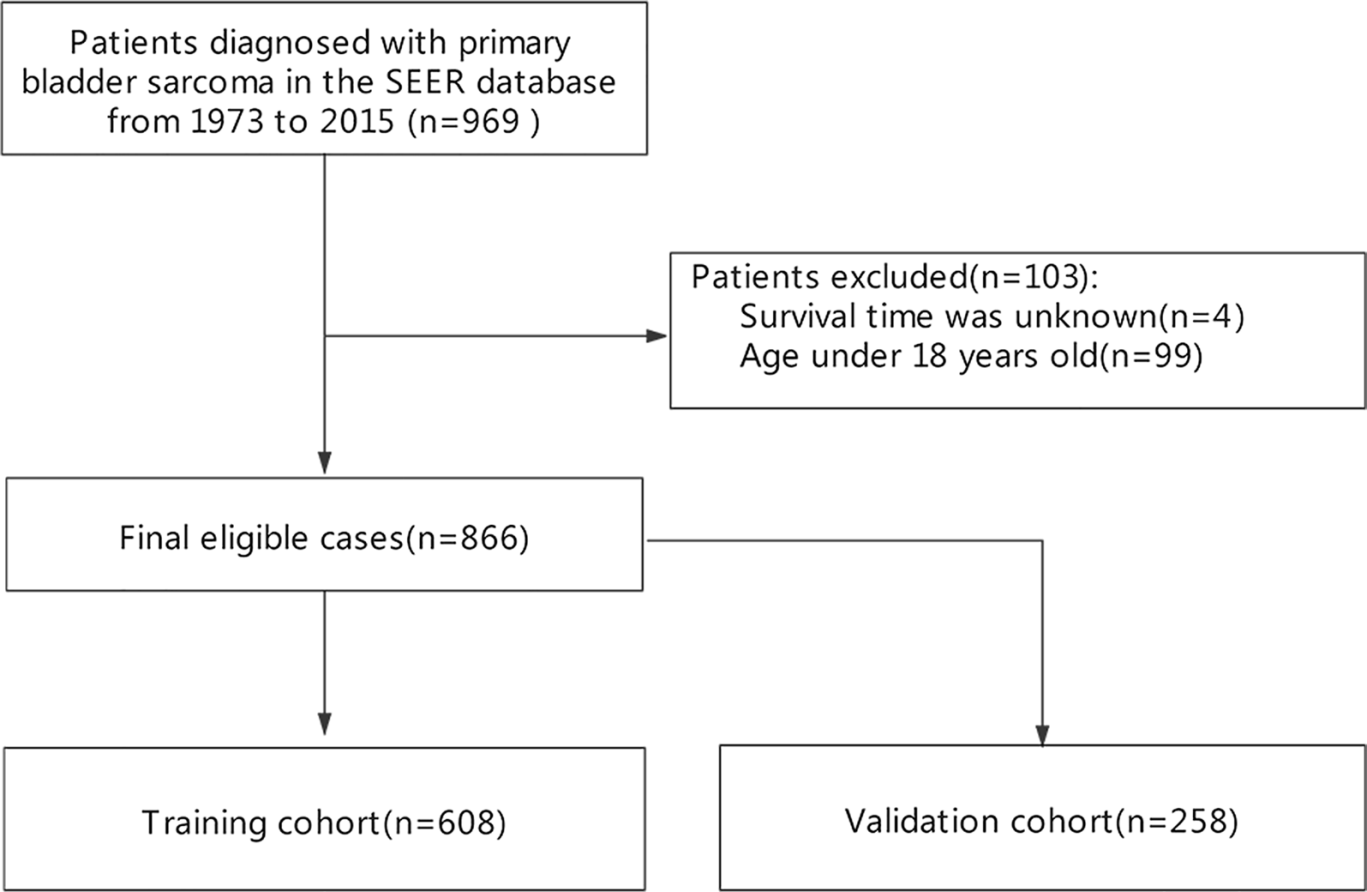
Flowchart showing the selection of patients

Data of the patients diagnosed with bladder sarcoma were extracted based on the International Classification of Tumor Diseases Third Edition (ICD-O-3). Inclusion criteria were as follows: (1) age at diagnosis > 18 years old; (2) histologically diagnosed as the first malignant tumor; (3) availability of complete demographic and sociological information, follow-up date, duration of survival (in months), and cause of death; (4) histological diagnosis of bladder sarcoma (ICD-O-3 Code: C67.0–C67.9); (5) adequate data regarding the patients' clinical stage, pathological grade, and other variables. After screening, 866 eligible PBS patients were finally included in the cohort. The process of data selection was shown in Fig. 1. The patients were randomly divided into two sets (training set, n = 608 and validation set, n = 258). Since SEER is a publicly available database, studies using the SEER database do not require ethical board approval and patient consent.

### Data collection

**Fig. 2:**
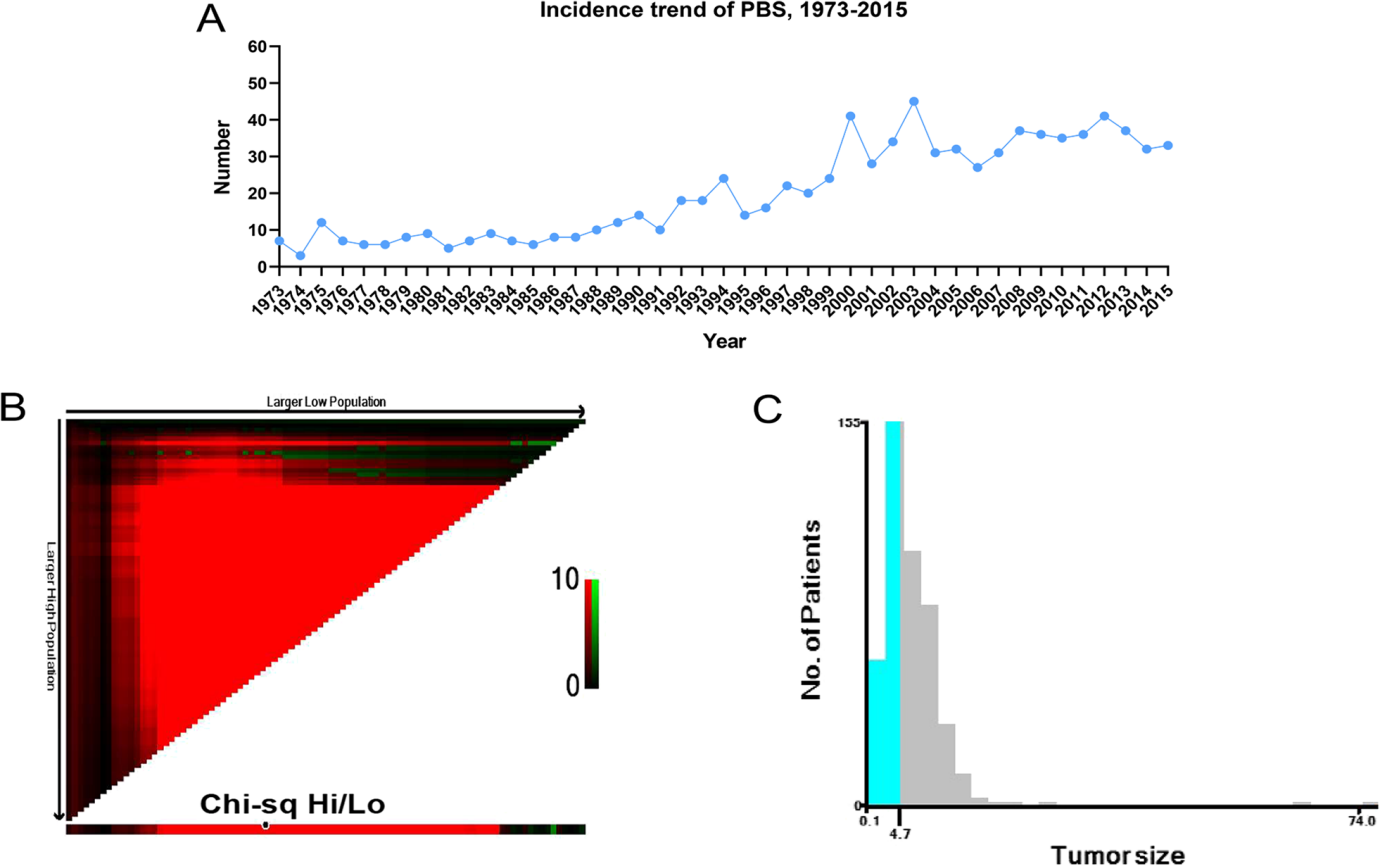
(**A**) An increase in the incidence of primary bladder sarcoma over the years. (**B**) X-tile analysis to determine the best cutoff value for tumor size in the entire cohort (**C**) The distributions of the number of patients based on x-tile analysis. OS, overall survival

Variables in the present study included age, sex, race, marital status, histological grade, pathological classification, pathological stage (TNM stage according to the American Joint Committee on Cancer staging system, third and sixth edition), tumor size, type of intervention such as radiation, chemotherapy and/or surgery, vital status, and duration of survival. As is shown in Fig. 2B, C, tumor size was divided into three categories by X-tile software version 3.6.1 (Rimm Lab, Yale School of Medicine, New Haven, CT, USA), which is a useful tool for finding optimal cutoff points of continuous data [15]. Overall survival (OS) was the primary endpoint. Duration of survival was calculated from the date of diagnosis to the date of last follow-up or until the date of death.

### Statistical analysis

Continuous variables were reported as median with range, and categorical variables as frequencies and proportions. The optimal cutoff values for age and tumor size were evaluated using the X-tile software. Univariate Cox regression analysis was performed to identify the significant prognostic factors. Afterwards, we incorporated them into the multivariable Cox proportional hazards regression models to further determine each variable’s independent association with survival outcomes. A nomogram for predicting the 1-, 3- and 5-year OS was constructed using the factors which remained significant in the multivariate Cox regression model. The predictive accuracy and discriminative ability of the nomogram were determined by the receiver operating characteristics (ROC) curves, the area under the curve (AUC), and Harrell’s concordance index (C-index). Calibration plots were generated to explore the performance characteristics of the nomogram at 1-, 3- and 5-year survival time. In addition, decision curve analysis (DCA), net reclassification improvement (NRI) and integrated discrimination improvement (IDI) were used to evaluate the clinical utility of the nomogram and to assess whether the model was more accurate than AJCC TNM staging system or not.

In addition, we calculated the total score of each patient based on the nomogram and constructed a risk stratification model accordingly, dividing the cohort into two different risk groups (low-risk group and high-risk group). The optimal cutoff value was analyzed by X-tile software. Kaplan–Meier survival analysis and Chi-square test were used to assess the significance of the difference in survival between the low- and high-risk groups.

All the analyses were performed with the statistical software package R 4.0.2 (http://www.R-project.org, The R Foundation, Vienna, Austria). Two-sided *P* values of less than 0.05 were considered statistically significant. All procedures performed in this study involving human participants conformed to the ethical standards described in the 1964 Helsinki declaration and its subsequent amendments.

## Results

### Patients’ baseline characteristics

Eight hundred and sixty-six patients with PBS in the SEER database met the study criteria and were included in this study. Figure 2A clearly showed an increase in the incidence of PBS over the years. Cohort demographics and tumor-related characteristics were described in Table 1. Most patients were male (529, 61.1%), white (743, 85.8%), and more than 80 years old (257, 29.7%). With regard to therapy, a majority of the patients underwent surgery (537, 62.0%), while fewer patients received radiation (112, 12.9%) or chemotherapy (132, 15.2%). Overall, the 1-, 3- and 5-year OS rates were 48.2%, 34.5% and 29.4%, respectively. Except for surgical treatment, there was no significant difference between the training and validation set (*P* > 0.05).

**Table 1:** Baseline demographic and clinical characteristics of patients with primary bladder sarcoma in the training cohort and validation cohort

Characteristics	Total cohort (n = 866)	Training cohort (n = 608)	Validation cohort (n = 258)	*P*-value
Total	866	608	258	
Age (IQR)	73.0 (60.2, 81.0)	73.0 (60.8, 81.0)	73.0 (60.2, 81.8)	0.963
*Age, n (%)*				0.517
18–29	27 (3.1)	21 (3.5)	6 (2.3)	
30–39	28 (3.2)	22 (3.6)	6 (2.3)	
40–49	56 (6.5)	33 (5.4)	23 (8.9)	
50–59	94 (10.9)	67 (11)	27 (10.5)	
60–69	156 (18.0)	109 (17.9)	47 (18.2)	
70–79	248 (28.6)	176 (28.9)	72 (27.9)	
≥ 80	257 (29.7)	180 (29.6)	77 (29.8)	
*Sex, n (%)*				1.000
Female	337 (38.9)	237 (39)	100 (38.8)	
Male	529 (61.1)	371 (61)	158 (61.2)	
*Race, n (%)*				0.189
White	743 (85.8)	530 (87.2)	213 (82.6)	
Black	90 (10.4)	58 (9.5)	32 (12.4)	
Other	33 (3.8)	20 (3.3)	13 (5)	
*Marital status, n (%)*				0.381
Married	484 (55.9)	342 (56.2)	142 (55)	
Single	353 (40.8)	249 (41)	104 (40.3)	
Unknown	29 (3.3)	17 (2.8)	12 (4.7)	
*Pathological classification, n (%)*			0.633	
Carcinosarcoma	408 (47.1)	296 (48.7)	112 (43.4)	
Leiomyosarcoma	207 (23.9)	141 (23.2)	66 (25.6)	
Sarcoma	84 (9.7)	59 (9.7)	25 (9.7)	
Spindle cell sarcoma	31 (3.6)	22 (3.6)	9 (3.5)	
Other	136 (15.7)	90 (14.8)	46 (17.8)	
*Histological grade, n (%)*				0.166
Well differentiated	22 (2.5)	16 (2.6)	6 (2.3)	
Moderately differentiated	38 (4.4)	29 (4.8)	9 (3.5)	
Poorly differentiated	202 (23.3)	151 (24.8)	51 (19.8)	
Undifferentiated	283 (32.7)	184 (30.3)	99 (38.4)	
Unknown	321 (37.1)	228 (37.5)	93 (36)	
*T-stage, n (%)*				0.124
Ta	68 (7.9)	55 (9)	13 (5)	
Tis	1 (0.1)	1 (0.2)	0 (0)	
T1	68 (7.9)	45 (7.4)	23 (8.9)	
T2	94 (10.9)	63 (10.4)	31 (12)	
T3	92 (10.6)	72 (11.8)	20 (7.8)	
T4	54 (6.2)	40 (6.6)	14 (5.4)	
Unknown	489 (56.5)	332 (54.6)	157 (60.9)	
*N-stage, n (%)*				0.286
No	476 (55.0)	329 (54.1)	147 (57)	
Yes	175 (20.2)	119 (19.6)	56 (21.7)	
Unknown	215 (24.8)	160 (26.3)	55 (21.3)	
*M-stage, n (%)*				0.326
No	593 (68.5)	407 (66.9)	186 (72.1)	
Yes	97 (11.2)	71 (11.7)	26 (10.1)	
Unknown	176 (20.3)	130 (21.4)	46 (17.8)	
*Tumor size, n (%)*				0.622
< 4.8	161 (18.6)	117 (19.2)	44 (17.1)	
≥ 4.8	291 (33.6)	199 (32.7)	92 (35.7)	
Unknown	414 (47.8)	292 (48)	122 (47.3)	
*Surgical treatment, n (%)*				0.023*
No	54 (6.2)	38 (6.2)	16 (6.2)	
Yes	537 (62.0)	360 (59.2)	177 (68.6)	
Unknown	275 (31.8)	210 (34.5)	65 (25.2)	
*Radiation*				0.360
No/Unknown, n (%)	754 (87.1)	534 (87.8)	220 (85.3)	
Yes	112 (12.9)	74 (12.2)	38 (14.7)	
*Chemotherapy, n (%)*				0.864
No/Unknown	734 (84.8)	514 (84.5)	220 (85.3)	
Yes	132 (15.2)	94 (15.5)	38 (14.7)	

*P < 0.05 indicating statistical significance

There were 709 events (deaths) in the cohort and the mean follow-up period was 42.1 months (median, 9.0 months; range 0–447 months).

### Screening for prognostic factors of OS

**Fig. 3:**
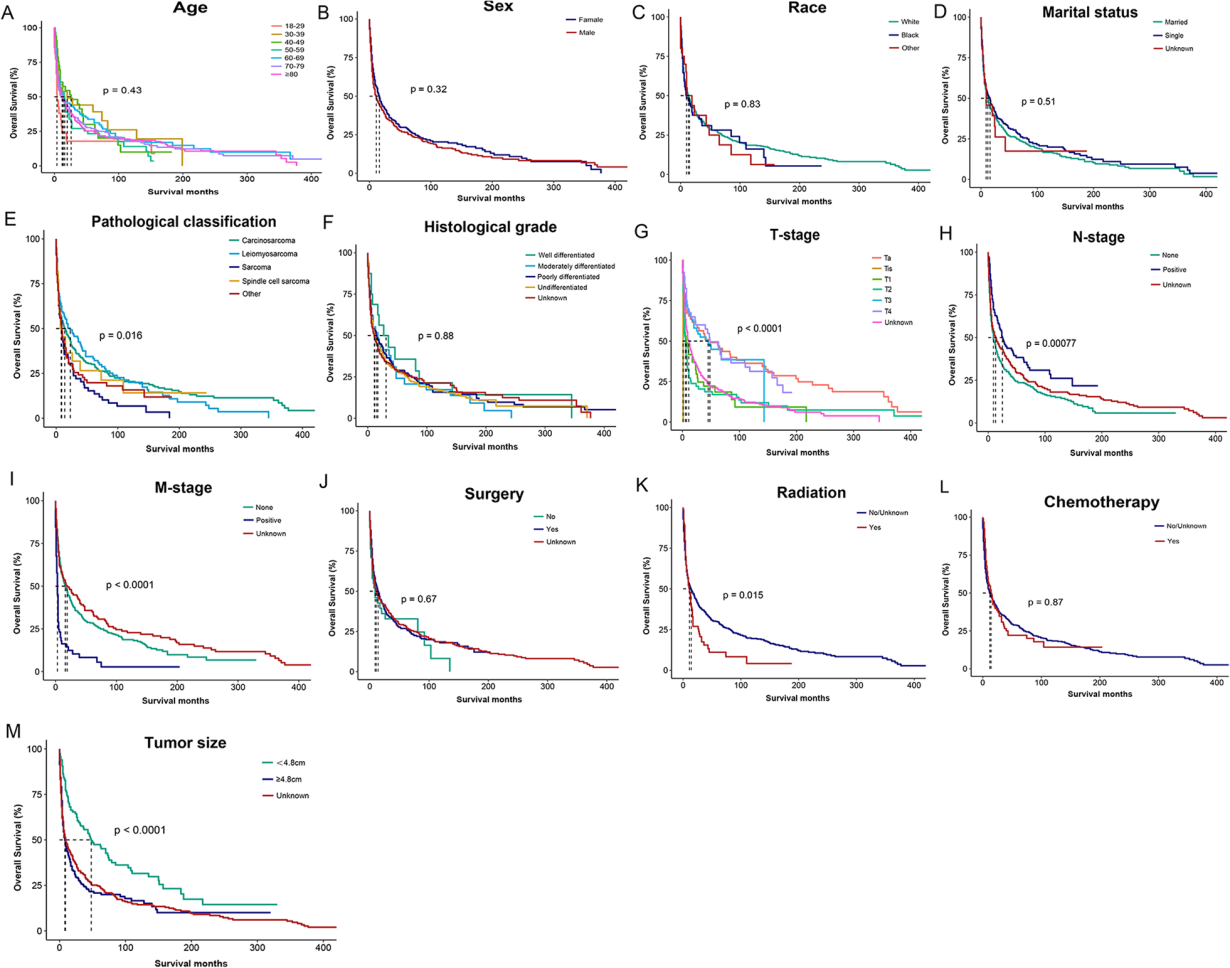
Kaplan–Meier curves of overall survival in patients with primary bladder sarcoma stratified by age (**A**), sex (**B**), race (**C**), marital status (**D**), pathological classification (**E**), histological grade (**F**), T-stage (**G**), N-stage (**H**), M-stage (**I**), tumor size (**J**), surgical treatment (**K**), radiation (**L**), and chemotherapy (**M**)

We conducted a univariable and multivariable Cox proportional hazards regression analysis to demonstrate the association between selected characteristics and oncological outcomes. Univariable Cox regression analysis identified seven variables (age, pathological classification, T-stage, N-stage, M-stage, tumor size, and radiation) as factors associated with a shorter OS. Multivariable Cox regression analysis indicated that statistically significant risk factors associated with a shorter OS included age, T-stage, N-stage, M-stage, and tumor size (Table 2). For example, patients with a tumor of a higher T stage or distant metastases may have a poor prognosis and worse cancer outcomes. Similarly, patients with a large tumor (≥ 4.8 cm) were more likely to have poor prognosis. Median OS in the training cohort was 11 months, with 1-, 3- and 5-year survival rates of 51.3%, 36.2%, and 30.8%, respectively. Kaplan–Meier analysis intuitively showed the different survival outcomes stratified according to the variables listed in Table 1 (Fig. 3A–M). Log-rank test showed significant differences in OS among subgroups in terms of pathological classification, T-stage, N-stage, M-stage, tumor size and radiotherapy (*P* < 0.05).

**Table 2:** Univariate and multivariate Cox regression analysis of included variables for OS in training cohort

Characteristics	Univariate analysis		Multivariate analysis	
	HR (95% CI)	*P*-value	HR (95% CI)	*P*-value
*Age*				
18–29	Reference		Reference	
30–39	0.53 (0.27, 1.03)	0.06	0.48 (0.24, 0.96)	0.039*
40–49	0.56 (0.31, 1.03)	0.062	0.43 (0.23, 0.80)	0.008*
50–59	0.71 (0.41, 1.2)	0.202	0.74 (0.42, 1.27)	0.274
60–69	0.58 (0.35, 0.97)	0.039*	0.55 (0.32, 0.94)	0.030*
70–79	0.65 (0.4, 1.06)	0.081	0.62 (0.37, 1.03)	0.063
≥ 80	0.66 (0.4, 1.07)	0.094	0.61 (0.37, 1.03)	0.063
*Sex*				
Female	Reference			
Male	1.1 (0.92, 1.32)	0.308		
*Race*				
White	Reference			
Black	1.07 (0.79, 1.46)	0.657		
Other	1.11 (0.68, 1.8)	0.681		
*Marital status*				
Married	Reference			
Single	0.9 (0.75, 1.08)	0.275		
Unknown	1.07 (0.61, 1.87)	0.806		
*Pathological classification*				
Carcinosarcoma	Reference		Reference	
Leiomyosarcoma	0.99 (0.79, 1.25)	0.927	0.79 (0.61, 1.03)	0.086
Sarcoma	1.55 (1.16, 2.07)	0.003*	1.36 (0.99, 1.88)	0.060
Spindle cell sarcoma	1.14 (0.7, 1.84)	0.602	0.94 (0.56, 1.60)	0.829
Other	1.3 (1, 1.69)	0.051	1.10 (0.83, 1.46)	0.510
*Histological grade*				
Well differentiated	Reference			
Moderately differentiated	1.35 (0.71, 2.57)	0.355		
Poorly differentiated	1.19 (0.69, 2.07)	0.534		
Undifferentiated	1.28 (0.74, 2.21)	0.381		
Unknown	1.25 (0.72, 2.15)	0.426		
*T-stage*				
Ta	Reference		Reference	
Tis	15.53 (2.11, 114.23)	0.007*	40.68 (5.17, 319.99)	< 0.001*
T1	2.08 (1.35, 3.21)	< 0.001*	2.20 (1.29, 3.75)	0.004*
T2	2.18 (1.48, 3.22)	< 0.001*	2.07 (1.33, 3.23)	0.001*
T3	1.0051 (0.65, 1.57)	0.982	1.15 (0.68, 1.96)	0.595
T4	1.02 (0.64, 1.62)	0.926	1.17 (0.67, 2.06)	0.574
Unknown	1.86 (1.36, 2.56)	< 0.001*	2.17 (1.45, 3.25)	< 0.001*
*N-stage*				
No	Reference		Reference	
Yes	0.62 (0.48, 0.8)	< 0.001*	0.74 (0.56, 0.97)	0.028*
Unknown	0.83 (0.68, 1.02)	0.084	1.03 (0.71, 1.50)	0.869
*M-stage*				
No	Reference		Reference	
Yes	2.7 (2.05, 3.55)	< 0.001*	2.74 (2.00, 3.75)	< 0.001*
Unknown	0.87 (0.69, 1.08)	0.205	0.85 (0.62, 1.19)	0.360
*Tumor size*				
< 4.8	Reference		Reference	
≥ 4.8	2 (1.51, 2.63)	< 0.001*	2.02 (1.52, 2.69)	< 0.001*
Unknown	1.86 (1.44, 2.41)	< 0.001*	2.11 (1.59, 2.81)	< 0.001*
*Surgical treatment*				
No	Reference			
Yes	0.84 (0.57, 1.23)	0.364		
Unknown	0.84 (0.56, 1.25)	0.396		
*Radiation*				
No/Unknown	Reference			
Yes	1.39 (1.06, 1.81)	0.017*	1.26 (0.94, 1.70)	0.118
*Chemotherapy*				
No/Unknown	Reference			
Yes	0.97 (0.75, 1.26)	0.842		

*P < 0.05 indicating statistical significance

### Prognostic nomogram construction for OS

**Fig. 4:**
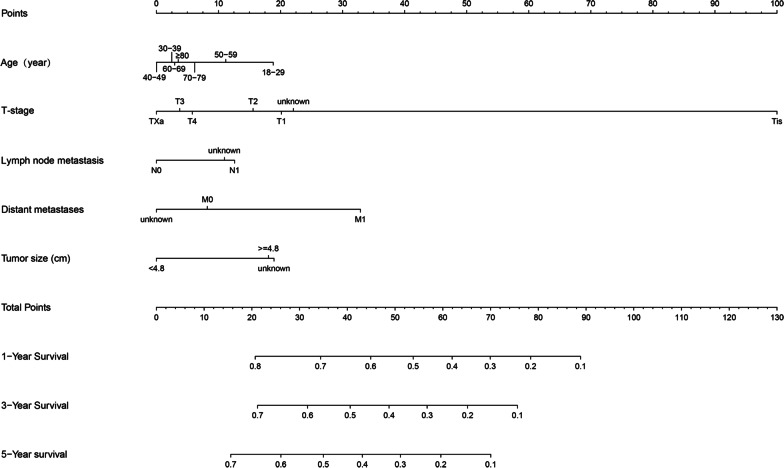
Nomogram model constructed using the independent prognostic factors predicting the 1-, 3- and 5-year OS for patients with PBS. OS, overall survival; PBS, primary bladder sarcoma

A nomogram was established based on the aforementioned significant prognostic factors for 1-, 3- and 5-year OS (Fig. 4), and then was validated internally. Each variable was given a score based on the hazard ratio. The total scores for each variable were added up and placed on the total subscale to obtain the probabilities of 1-, 3- and 5-year OS. As shown in Additional file 1: Figure S1, using the nomogram, it could be concluded that a 70-year-old patient with T2N0M0 and a tumor size of 5 cm would score 47.5 points, which means that the patient has about 57.5%, 42%, and 34.5% survival probability 1, 3, and 5 years after the diagnosis, respectively.

### Calibration and validation of the nomogram

On the training cohort, the C-index of the nomogram for 1-, 3- and 5-year OS prediction were 0.675 [95% confidence interval (CI): 0.648–0.702], 0.670 (95% CI: 0.642–0.697) and 0.671 (95% CI: 0.643–0.698), respectively. On the validation cohort, the C-indexes at 1-, 3-, and 5-year were 0.701 (95% CI 0.674–0.728), 0.684 (95% CI 0.657–0.711), and 0.679 (95% CI 0.651–0.706), respectively. The data indicated brilliant discrimination ability of the nomogram.

**Fig. 5:**
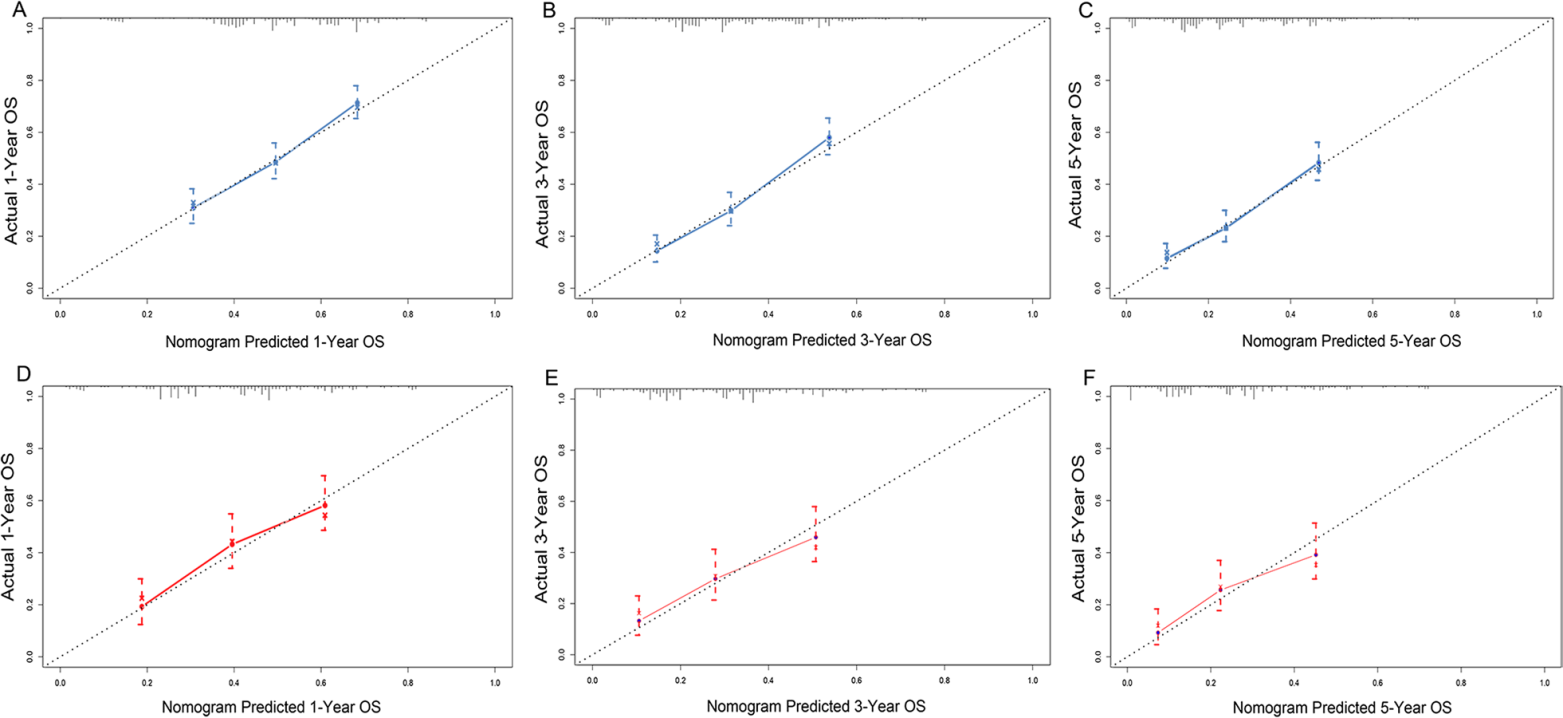
Calibration plots for the nomogram. Calibration plots of 1-year (**A**), 3-year (**B**), and 5-year (**C**) OS in the training cohort; Calibration plots of 1-year (**D**), 3-year (**E**), and 5-year (**F**) OS in the validation cohort. OS, overall survival

Meanwhile, the calibration plots of the training cohort for 1-, 3- and 5-year OS displayed consistency between the observed and predicted results (Fig. 5A–C). Similarly, the calibration plots of the 1-, 3- and 5-year OS were well calibrated in the validation cohort (Fig. 5D–F).

### Comparison of the nomogram and AJCC TNM staging system

**Fig. 6:**
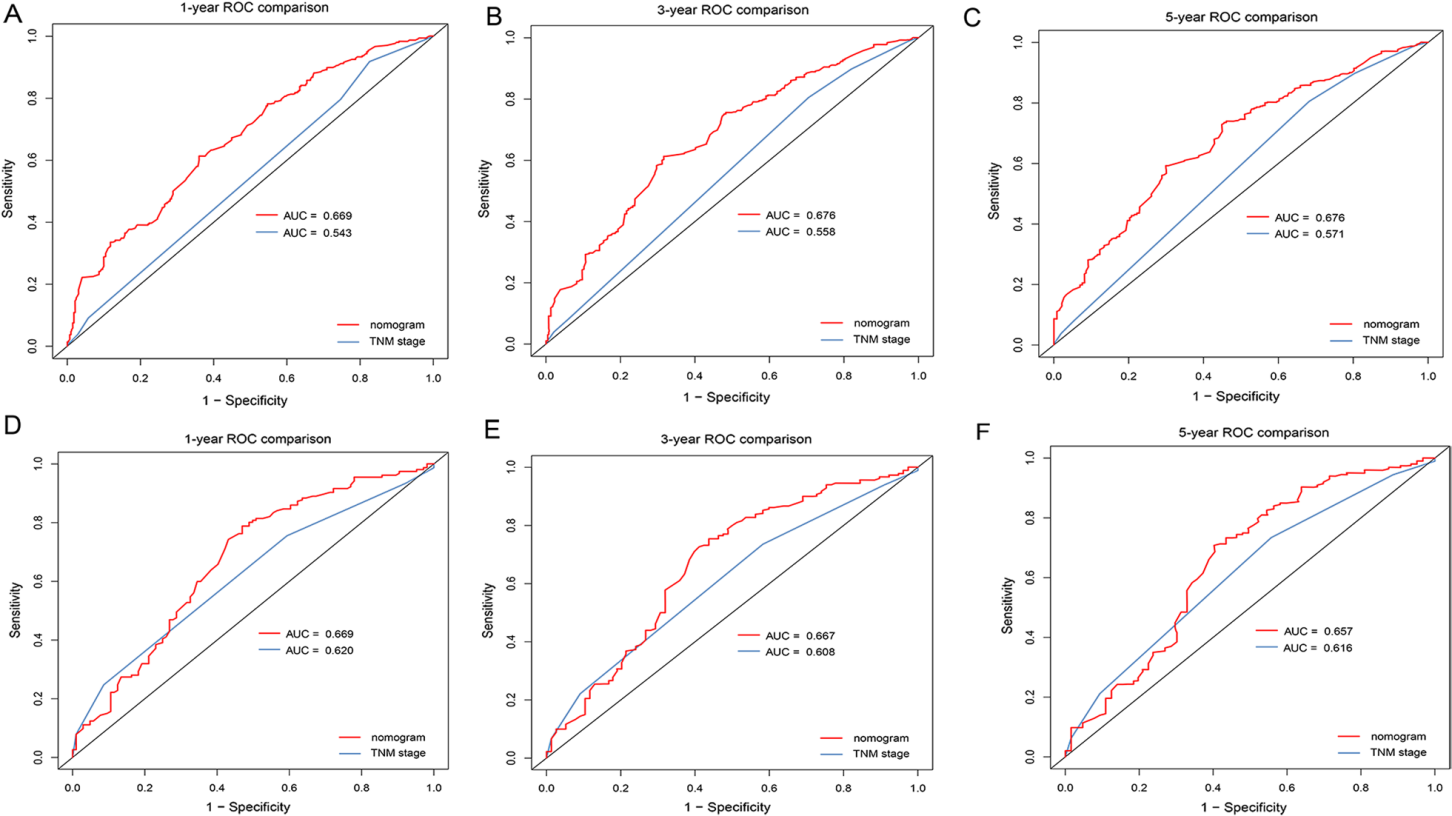
ROC curves of the nomogram for OS compared with TNM staging. ROC curves comparation of the nomogram and TNM staging for 1-year (**A**), 3-year (**B**) and 5-year (**C**) OS in the training cohort. ROC curves comparation of the nomogram and TNM staging for 1-year (**D**), 3-year (**E**) and 5-year (**F**) OS in the validation cohort. AUC: area under the curve; ROC, receiver operating characteristic; OS, overall survival

ROC curves analysis showed that the AUCs of the nomogram for 1-, 3- and 5-year OS were better than those of TNM stage both in the training (Fig. 6A–C) and validation cohort (Fig. 6D–F).

**Fig. 7:**
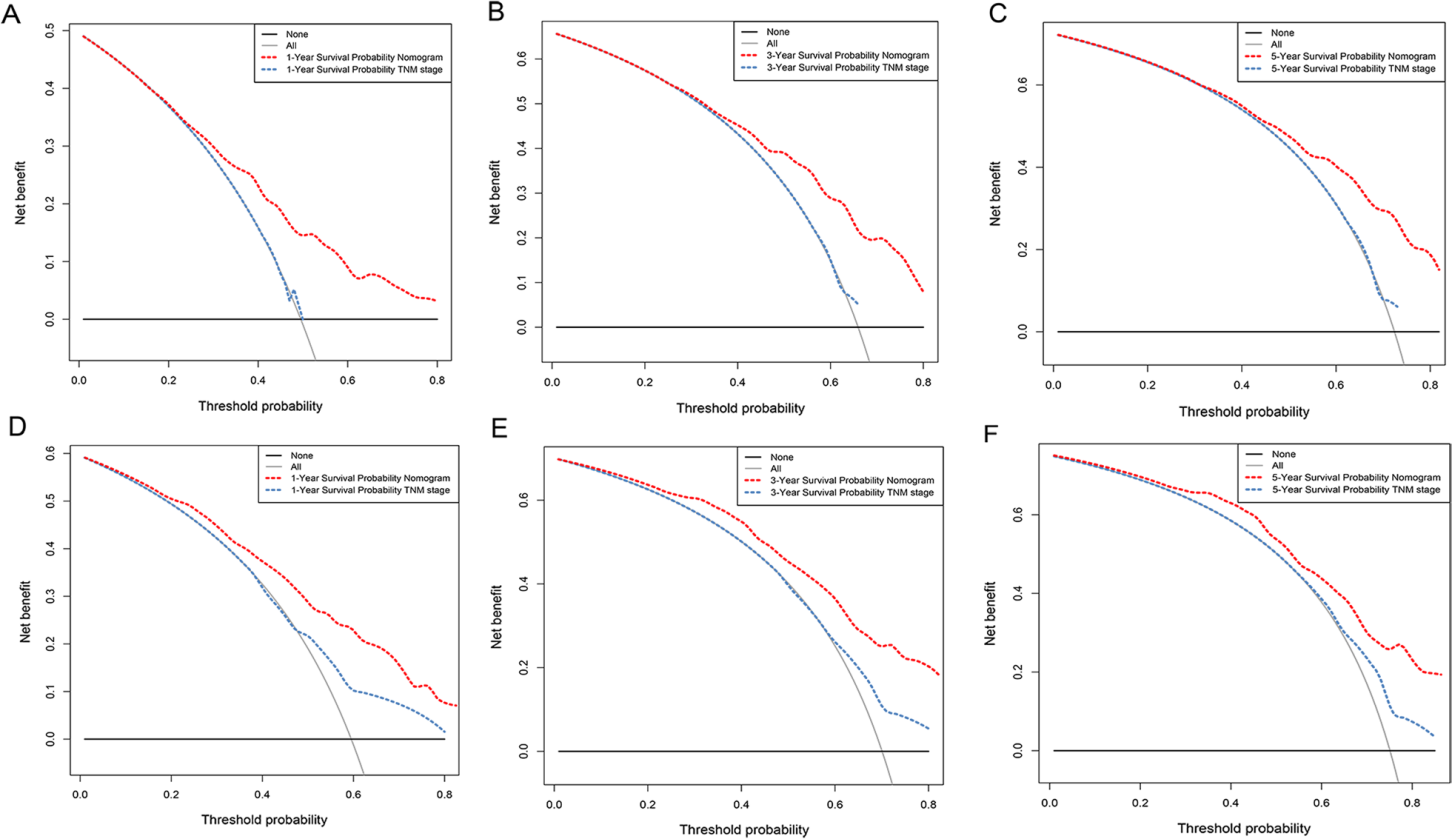
DCA of the nomogram and AJCC TNM staging for 1-year (**A**), 3-year (**B**) and 5-year (**C**) OS in training cohort, and for 1-year (**D**), 3-year (**E**) and 5-year (**F**) OS in the validation cohort. The red dashed line represents the nomogram. The blue dashed line represents AJCC TNM stage. OS, overall survival; DCA, decision curve analyses; AJCC, American Joint Committee on Cancer

DCA analysis showed that compared with the AJCC TNM staging system, the net benefit of the new nomogram is significantly increased and has a wide range of threshold probabilities both in the training (Fig. 7A–C) and validation cohort (Fig. 7D–F). This indicated that the nomogram can be more beneficial in the clinical application of predicting individual survival outcomes than TNM staging system.

In the NRI and IDI analyses, the nomogram performed better than TNM staging system (Table 3). In the training cohort, the 1-, 3- and 5-year NRI of the nomogram compared to TNM staging system was 15.3% (*p* < 0.01), 21.0% (*p* < 0.01) and 21.2% (*p* < 0.01), respectively. And the 1-, 3- and 5-year IDI of the nomogram compared to TNM staging system was 3.3% (*p* < 0.01), 4.6% (*p* < 0.01) and 4.4% (*p* < 0.01), respectively. In the validation cohort, the 1-, 3- and 5-year NRI of the nomogram compared to TNM staging system was 19.8% (*p* < 0.01), 16.5% (*p* = 0.02) and 17.9% (*p* = 0.01), respectively. And the 1-, 3- and 5-year IDI of the nomogram compared to TNM staging system was 7.9% (*p* < 0.01), 7.9% (*p* < 0.01) and 8.6% (*p* < 0.01), respectively.

**Table 3:** NRI and IDI of the nomogram in survival prediction for PBS patients compared with TNM staging

Index	Training cohort			Validation cohort		
	Estimate	95% CI	*P*-value	Estimate	95% CI	*P*-value
*NRI (vs. TNM staging)*						
For 1-year OS	0.153	0.048–0.238	< 0.01*	0.198	0.072–0.367	< 0.01*
For 3-year OS	0.210	0.084–0.297	< 0.01*	0.165	0.026–0.343	0.02*
For 5-year OS	0.212	0.084–0.304	< 0.01*	0.179	0.029–0.362	0.01*
*IDI (vs. TNM staging)*						
For 1-year OS	0.033	0.015–0.058	< 0.01*	0.079	0.037–0.149	< 0.01*
For 3-year OS	0.046	0.019–0.081	< 0.01*	0.079	0.033–0.151	< 0.01*
For 5-year OS	0.044	0.017–0.081	< 0.01*	0.086	0.032–0.169	< 0.01*

NRI, net reclassification improvement; IDI, integrated discrimination improvement; PBS, primary bladder sarcoma; OS, overall survival

*P < 0.05 indicating statistical significance

### Ability of nomogram to stratify patient risk

**Fig. 8:**
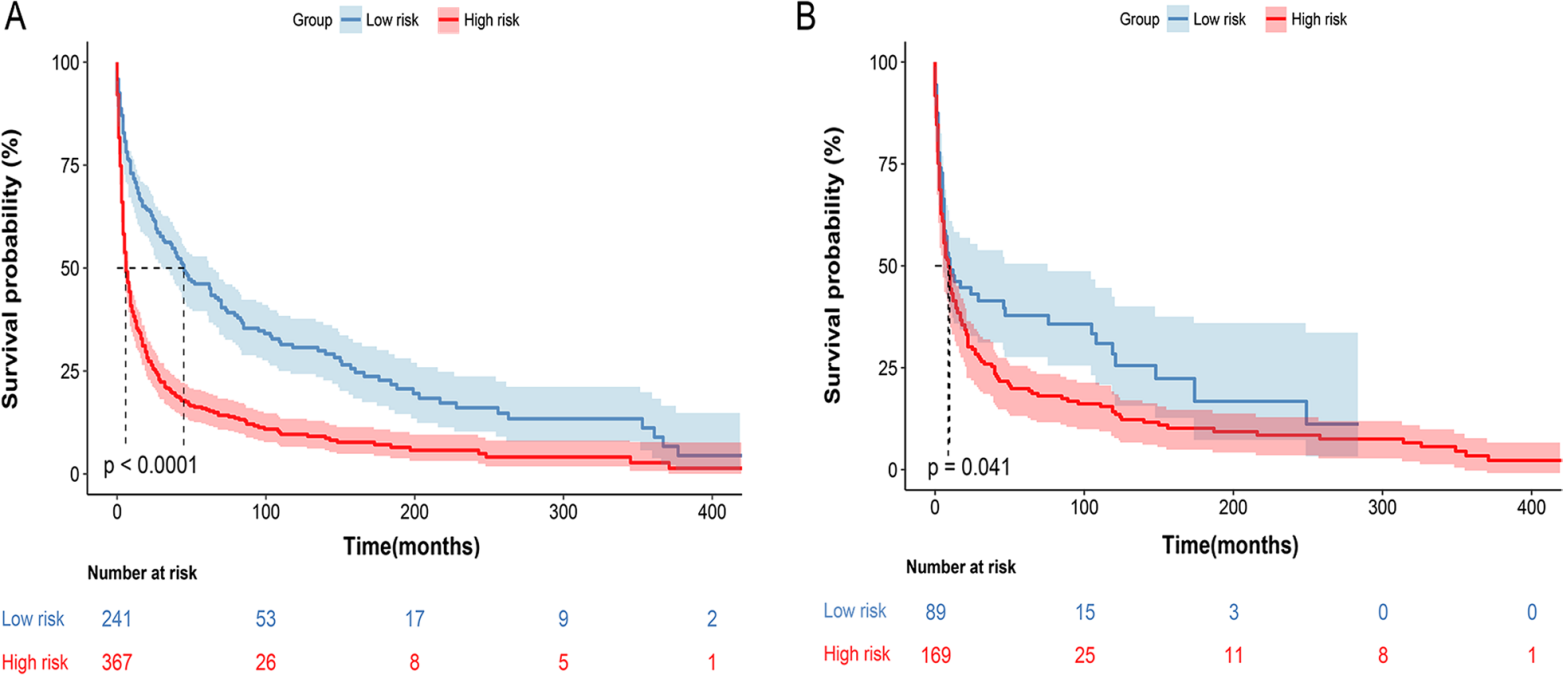
Kaplan–Meier survival analyses to test the risk stratification system within the training (**A**) and the validation cohort (**B**). The blue line represents low-risk group, and the red line represents high-risk group

The cut-off point between the high-risk and low-risk cohorts was determined as 47 by X-tile analysis, and the 608 patients in the training cohort were divided into a high-risk group (total score > 47) and a low-risk group (total score ≤ 47) based on this cut-off value. By Kaplan–Meier analysis (Fig. 8A), 367 high-risk patients had significantly more severe OS than 241 low-risk patients (*p* < 0.0001). Application of this cutoff value also significantly distinguished the high-risk and low-risk groups in the validation cohort (*p* = 0.041) (Fig. 8B).

## Discussion

Around 430,000 new cases of bladder cancer are diagnosed worldwide each year, and this cancer is associated with high morbidity and mortality [16]. As a rare subtype of bladder cancer, the incidence rate of PBS is very low, due to which tumor progression is not well-understood. The clinical significance and biologic behavior of this subtype of bladder cancer warrant additional investigation. In the present study, vast amount of data collected by the SEER program was utilized to examine the largest series of PBS cases reported to date. This study was the first attempt to date to use the SEER database to build a predictive model for better understanding the survival outcomes of PBS at a population level.

Several unique features of PBS distinguish it from urothelial bladder cancer, and are worth mentioning. PBS has multiple types, and leiomyosarcoma and rhabdomyosarcoma account for 50% and 20% of PBS cases, respectively [17]. Other histological types of PBS include osteosarcoma, angiosarcoma, myxoid liposarcoma, fibrosarcoma, malignant fibroblastic tumor, carcinosarcoma, and plexiform sarcoma [18]. Besides, previous studies have emphasized the poor prognosis of PBS. Rosser et al. [19] reported one of the largest series of 36 adult PBS patients treated between 1986 and 1998. The 5-year disease-specific survival rate was 62.0%. In another systematic review and meta-analysis that included by far the largest number of cases containing 210 patients with PBS between 1970 and 2018, Zieschang et al. [20] determined a 5-year cancer-specific cumulative mortality rate of 38% for patients with PBS. We found that the prognosis of PBS was poor and did not change significantly over decades. In addition, previous studies have shown that the vast majority of PBS patients are elderly men with significant pain. However, hematuria is rare, which is very different from the presentation of urothelial tumors [21, 22]. The treatment of this rare tumor was challenging. The most promising treatment options still seemed to be radical cystectomy over the past few decades, possibly supplemented by chemotherapy or radiotherapy. However, as time migrated and technology developed, partial cystectomy was one of the surgical options available for smaller tumors.

To date, there are no large, prospective, randomized controlled trials on PBS treatment strategies worldwide. Therefore, it is unknown whether the survival rate of the patients depends upon the type of treatment modality. Due to the specificity of PBS, there is no specially designed or widely accepted grading system or prediction model. However, early identification of the disease and effective treatment can significantly improve the prognosis. Hence, efforts to establish predictive models to promote the management of these patients according to their individualized prognosis are justified. In our study, we established and validated a novel predictive tool based on age, tumor stage, lymph node status, distant metastasis, and tumor size that can be used to guide clinical practice.

In recent years, nomograms, developed using the SEER database, have been widely used to predict the prognosis of various malignancies, such as Ewing sarcoma, penile carcinoma, and cardiac sarcoma [23–25]. Our current study was the first to construct a well‐validated nomogram including several clinical features and risk scores for patients with PBS, which can predict the clinical prognosis of PBS intuitively and effectively. The variables in the nomogram were independent factors affecting OS, which led to a better prediction of the survival of patients with PBS. Using this nomogram, we will be able to predict the future survival rate of the patients more accurately. Although the C-indexes and AUCs of the nomogram in the training and validation cohort were not high enough, the predictive ability of the model was more accurate than using the current TNM staging to predict the prognosis. Further DCA, NRI and IDI analyses demonstrated its clear clinical application advantages over the TNM staging system. A risk stratification model based on this nomogram can effectively classify patients in the training or validation cohort into two risk groups (high risk and low risk) and OS can be distinguished. The results of this study could be particularly helpful in predicting postoperative survival of the PBS patients.

Our nomogram is innovative and reasonable in the following aspects: firstly, to the best of our knowledge, this study is the first to attempt to develop a prognostic nomogram for OS of the PBS patients using population-based data, which can provide individualized treatment guidance. Secondly, variables like age, T/N/M stage, and tumor size were used to develop this nomogram. It is worth noting that in order to maintain the integrity of the data, factors containing negative or unknown information were also included in the analysis. Taking tumor size as an example, 414 cases (47.8%) had unknown tumor size. In addition, according to the multivariate Cox regression analysis and nomogram, unknown tumor size seemed to reduce the survival rate, which may be due to the heterogeneity of these tumors. However, we could not exclude this group of patients from the study; otherwise, potential selection bias could have been introduced. On the contrary, using continuous queues and complete information can guarantee more accurate results. Finally, the nomogram based on the SEER database was able to predict the prognosis of PBS. ROC curve, DCA, NRI and IDI analyses of this study showed that the nomogram could predict the death of patients with PBS more accurately, which has clinical applicability. The results of the internal validation of nomogram prediction were found to be consistent.

There are some limitations in our research. Firstly, this is a population-based retrospective analysis using the SEER database, which does not include certain important variables, such as preoperative laboratory results and socio-economic status, which are also reported related to the prognosis of patients with bladder malignant tumor [26]. Secondly, although the selection bias is avoided to some extent, a large amount of information is missing in the SEER database, which may have affected the prediction model. Thirdly, this study only considered OS as the primary endpoint and did not include disease-specific survival, which may partly limit the broad application of the results. Fourthly, there are no data available for external validation and due to the nature of retrospective studies; the nomogram needs to be validated for a prospective cohort.

## Conclusion

In this study, we developed and validated a nomogram to predict the OS rate of patients with PBS, and it showed consistent reliability and clinical applicability. The nomogram can assist clinicians in evaluating the risk factors for poor prognosis in patients with PBS and formulating optimal individualized treatment strategies. However, further evaluation in other patient groups is needed to establish the external validity of our findings.

## Supplementary Information

**Additional file 1: Figure S1.** Nomogram is used to evaluate a 70-year-old patient with T2N0M0 and a tumor size of 5 cm. Based on the total score, the survival probability 1-year, 3-year, and 5-year of the patient is 57.5%, 42%, and 34.5%, respectively.

## Data Availability

The datasets analyzed during the current study are available in the SEER*Stat software (version 8.3.6, download from https://seer.cancer.gov/data/options.html). A registration form needs to be completed before using and filter criteria need to be added. The datasets are also available from the corresponding author on reasonable request.

## Abbreviations

OS: Overall survival
PBS: Primary bladder sarcoma
CI: Confidence interval
SEER: Surveillance, Epidemiology, and End Results
DCA: Decision curve analysis
ROC: Receiver operating characteristics
AUC: The area under the curve
C-index: Concordance index
AJCC: American Joint Committee on Cancer
NRI: Net reclassification improvement
IDI: Integrated discrimination improvement
