# Supplementary figures and images for: Development and validation of a prognostic nomogram for predicting overall survival in patients with primary bladder sarcoma: a SEER-based retrospective study
Source: BMC Urol. 2021 Nov 25;21:162. doi: 10.1186/s12894-021-00929-x (PMC8614032; doi:10.1186/s12894-021-00929-x)

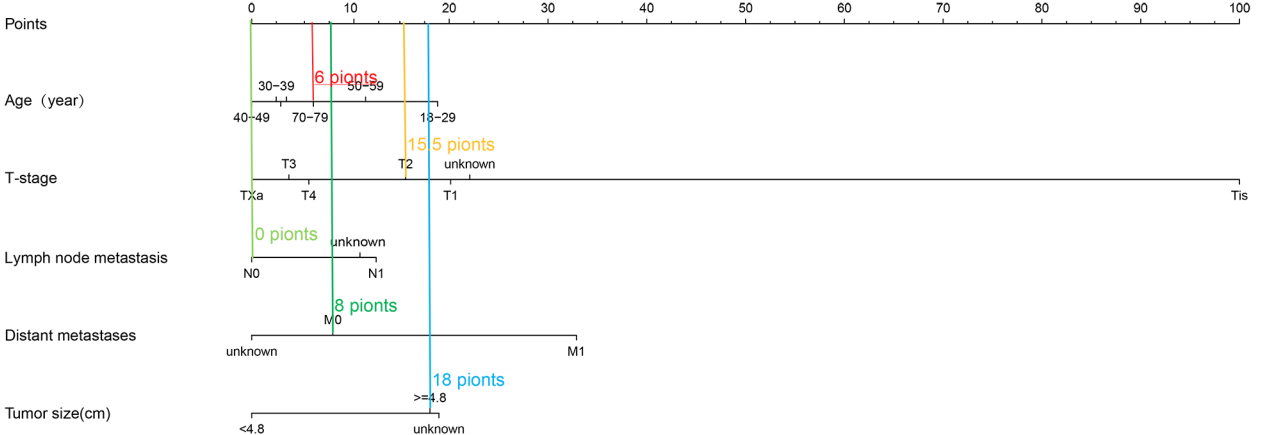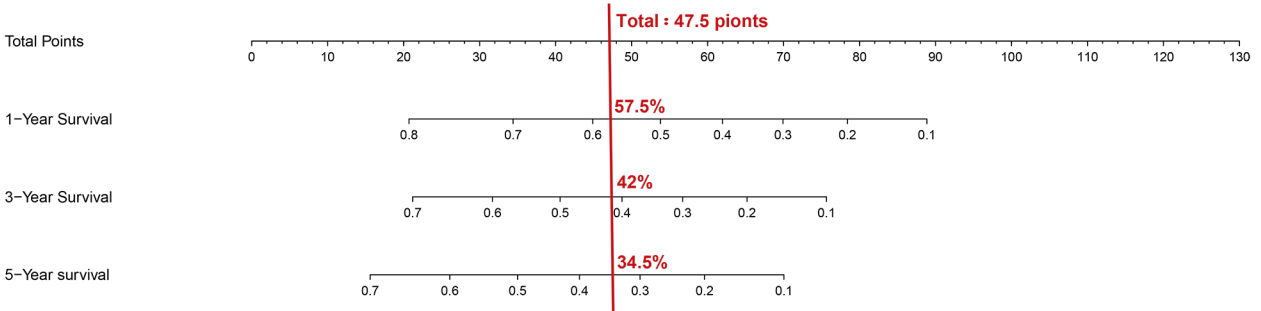

Supplement: Supplementary file 1 — Additional file 1: Figure S1. Nomogram is used to evaluate a 70-year-old patient with T2N0M0 and a tumor size of 5 cm. Based on the total score, the survival probability 1-year, 3-year, and 5-year of the patient is 57.5%, 42%, and 34.5%, respectively. [file 12894_2021_929_MOESM1_ESM.pdf]
